# The therapeutic value of XL388 in human glioma cells

**DOI:** 10.18632/aging.103791

**Published:** 2020-11-06

**Authors:** Shan Zhong, Jun Xue, Jiao-Jiao Cao, Bomin Sun, Qing-Fang Sun, Liu-Guan Bian, Liang-Yun Hu, Si-Jian Pan

**Affiliations:** 1 Department of Neurosurgery, Rui-Jin Hospital, Shanghai Jiao-Tong University School of Medicine, Shanghai, China; 2 Department of Stereotactic and Functional Neurosurgery, Rui-Jin Hospital, Shanghai Jiao-Tong University School of Medicine, Shanghai, China

**Keywords:** glioma, mTOR, Akt, XL388, MAFG

## Abstract

XL388 is a highly efficient and orally-available ATP-competitive PI3K-mTOR dual inhibitor. Its activity against glioma cells was studied here. In established and primary human glioma cells, XL388 potently inhibited cell survival and proliferation as well as cell migration, invasion and cell cycle progression. The dual inhibitor induced significant apoptosis activation in glioma cells. In A172 cells and primary human glioma cells, XL388 inhibited Akt-mTORC1/2 activation by blocking phosphorylation of Akt and S6K1. XL388-induced glioma cell death was only partially attenuated by a constitutively-active mutant Akt1. Furthermore, it was cytotoxic against Akt1-knockout A172 glioma cells. XL388 downregulated MAF bZIP transcription factor G (MAFG) and inhibited Nrf2 signaling, causing oxidative injury in glioma cells. Conversely, antioxidants, n-acetylcysteine, pyrrolidine dithiocarbamate and AGI-106, alleviated XL388-induced cytotoxicity and apoptosis in glioma cells. Oral administration of XL388 inhibited subcutaneous A172 xenograft growth in severe combined immunodeficient mice. Akt-S6K1 inhibition and MAFG downregulation were detected in XL388-treated A172 xenograft tissues. Collectively, XL388 efficiently inhibits human glioma cell growth, through Akt-mTOR-dependent and -independent mechanisms.

## INTRODUCTION

Glioma is the most common primary brain tumor and among the most aggressive of human cancers [1, 2]. Over 10, 000 people are diagnosed with glioma each year in the United States, mostly with high-grade tumors [3]. The average survival of glioma patients is less than a year after initial diagnosis [1, 2]. Significant progress has been made in glioma treatments, including neurosurgical resection, radiation and chemotherapy [1, 2]. However, the five-year survival rate remains very disappointing [1, 2]. It is therefore urgent to explore more efficient targeted therapies [4].

Due to epidermal growth factor receptor (EGFR) amplification, PTEN depletion and possible other mutations, overactivation of PI3K-Akt-mammalian target of rapamycin (mTOR) cascade is commonly detected in human glioma [5, 6]. It is associated with tumorigenesis, progression and poor prognosis [7, 8]. This cascade is therefore an important therapeutic target [5, 6]. mTOR exists in at least two distinct kinase complexes, mTOR complex 1 (mTORC1) and mTOR complex 2 (mTORC2). Both of which are essential for cell growth, proliferation, survival, apoptosis-resistance and angiogenesis [9–11]. mTOR1 is rapamycin-sensitive and is composed of mTOR, Raptor, PRAS40 and several other components. mTORC1 phosphorylates S6K1 and 4E-BP1 [9–11]. The rapamycin-insensitive mTORC2 is assembled by mTOR, Rictor, Sin1 and Protor, and serves as an upstream kinase of Akt at Ser-473 [9–11]. Several mTOR small molecule inhibitors are currently in clinical trials and may have some activity against human glioma [5, 6].

Our group has shown that overactivation of mTOR signaling is important for glioma cell progression [12–15]. We show that tetraspanin 8 (Tspan8) can form a complex with Rictor, which is required for mTORC2 activation and glioma cell migration [15]. Furthermore, GSK621, an AMP-activated protein kinase (AMPK) activator, inhibited mTORC1 activation and glioma cell survival [14]. Recently, we found that melanoma antigen A6 (MAGEA6) silencing restored AMPKα1 expression, causing mTORC1 inhibition and glioma cell apoptosis [13]. Further, LncRNA THOR (Lnc-THOR) silencing inhibited glioma cell survival by depleting MAGEA6 and inhibiting mTOR signaling [12]. Recent research has characterized a highly efficient and orally-available ATP-competitive PI3K-mTOR dual inhibitor, XL388 [16]. It has displayed anti-cancer activity in several preclinical cancer studies [16–18]. Its activity against human glioma cells and underlying mechanisms are largely unknown.

The nuclear factor erythroid-derived 2-like 2, Nrf2, is an essential transcription factor responsible for expression of anti-oxidant genes [19–23]. Nrf2 forms a heterodimer with MAF bZIP transcription factor G (MAFG) that binds to antioxidant response element (ARE). It will promote transcription of detoxification genes and reactive oxygen species (ROS)-scavenging genes, including *HO1* (*HMOX1), NQO1* and others [19–21]*.* Here we will show that XL388 downregulated MAFG, causing Nrf2 signaling inhibition and ROS production in glioma cells.

## RESULTS

### XL388 potently inhibits glioma cell survival, proliferation, migration, invasion and cell cycle progression

**Figure 1:**
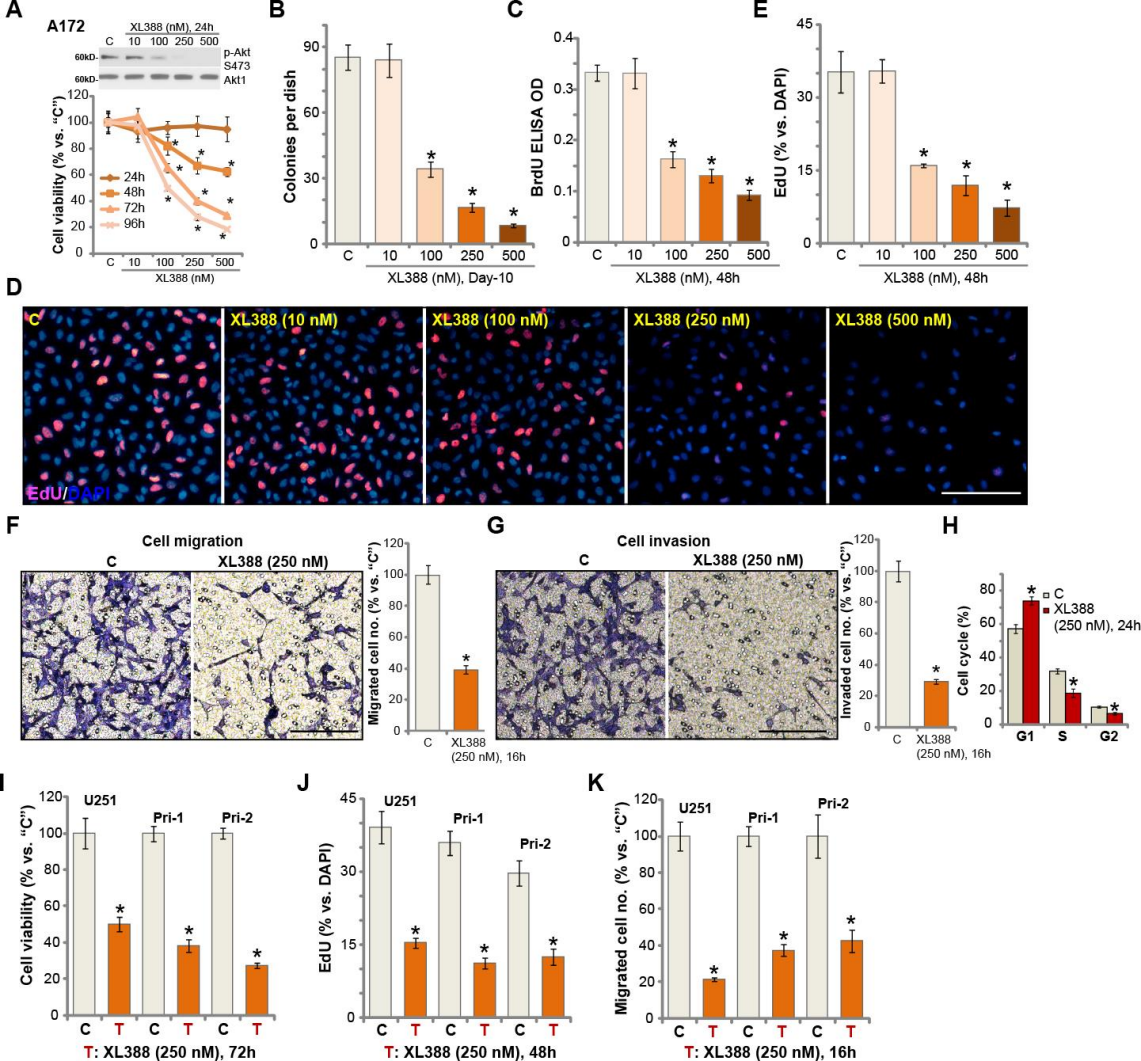
**XL388 potently inhibits glioma cell survival, proliferation, migration, invasion and cell cycle progression.** A172 cells (**A**–**H**), U251MG cells (“U251”) (**I**–**K**) and primary human glioma cells (“Pri-1/Pri-2”) (**I**–**K**) were treated with applied concentrations of XL388 or the vehicle control (“C”, same for all Figures), and cultured for applied time periods, then cellular functions including cell survival (**A**, **B** and **I**), proliferation (**C**–**E**, and **J**), migration (**F** and **K**), invasion (**G**) and cell cycle progression (**H**) were tested by the indicated assays. Results were quantified. Expression of listed proteins was shown (**A**). Data were presented as mean ± SD (n=5). * p <0.05 vs. “C” cells. Experiments in this figure were repeated three times, and similar results were obtained. Bar= 100 μm (**D**, **F** and **G**).

A172 glioma cells ([12, 13]) were cultured in complete medium (containing 10% FBS) and treated with different concentrations of XL388 (from 10-500 nM). Cells were further cultured for 24-96h. Analyzing cell viability, by CCK-8 assays, demonstrated that XL388 inhibited A172 cell viability in a dose-dependent manner (Figure 1A). The PI3K-mTOR dual inhibitor displayed a time-dependent response as well. XL388 (at 100-500 nM) required at least 48h to exert a significant anti-survival activity (Figure 1A). In A172 cells, XL388-induced viability reduction lasted for at least 96h (Figure 1A). It was ineffective at lowest concentration tested (10 nM) (Figure 1A). XL388 dose-dependently inhibited Akt activation (Akt Ser-473 phosphorylation) in A172 cells (Figure 1A). The colony formation assay results, Figure 1B, demonstrated that XL388 dose-dependently decreased the number of viable A172 cell colonies. XL388 at 100-500 nM significantly inhibited A172 cell proliferation, BrdU incorporation (Figure 1C) and nuclear EdU staining (Figure 1D and 1E). XL388 at 10 nM was again ineffective (Figure 1C–1E). In these assays, the IC-50 of XL388 is close to 250 nM (Figure 1A–1E) and this concentration was selected for following studies.

By applying “Transwell” and “Matrigel Transwell” assays, we show that XL388 (250 nM) inhibited A172 cell migration (Figure 1F) and invasion (Figure 1G) *in vitro*. The quantitative analysis demonstrated that it significantly reduced the number of migrated (Figure 1F) and invaded (Figure 1G) A172 cells. Analyzing cell cycle, by the propidium Iodide (PI)-FACS assay, show that XL388 (250 nM) treatment in A172 cells led to an increase in G1 phase cells, but decreases in S-/G2-phase cells (Figure 1H), indicating G1-S arrest in XL388-treated cells. For the cell migration/invasion and cell cycle analyses, cells were treated with XL388 (250 nM) for 24h or less, when no significant cytotoxicity or proliferation inhibition were detected (Figure 1A). Therefore, XL388 potently inhibited A172 cell viability, proliferation, migration, invasion and cell cycle progression. The potential effect of XL388 in other human glioma cells was studied as well. As described early, the primary human glioma cells, Pri-1/Pri-2 (derived from two different patients [12–14]), as well as the established U251 glioma cells, were cultured and treated with XL388 (250 nM). XL388 treatment resulted in significant inhibition of cell viability (CCK-8 OD, Figure 1I), proliferation (EdU incorporation, Figure 1J) and migration (“Transwell” assays, Figure 1K).

### XL388 induces significant apoptosis activation in glioma cells

**Figure 2:**
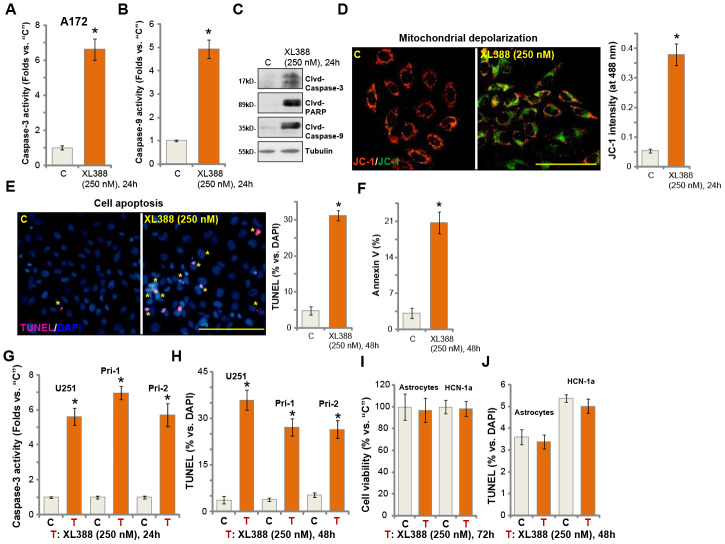
**XL388 induces significant apoptosis activation in glioma cells.** A172 cells (**A**–**F**), U251MG (“U251”) (**G** and **H**) and primary human glioma cells (“Pri-1/Pri-2”) (**G** and **H**) as well as the primary human astrocytes (“Astrocytes”) and HCN-1a neuronal cells (**I** and **J**) were treated with XL388 (250 nM), and cultured for applied time periods, then cell apoptosis was analyzed by the mentioned assays (**A**–**H** and **J**), with cell viability tested by CCK-8 assay (**I**). Data were presented as mean ± SD (n=5). * p <0.05 vs. “C” cells. Experiments in this figure were repeated three times, and similar results were obtained. Bar= 100 μm (**D** and **E**).

In human cancer cells, proliferation inhibition and cell cycle arrest could induce cell apoptosis. We therefore studied the potential effect of XL388 on glioma cell apoptosis. In A172 cells, the caspase-3 activity (Figure 2A) and the caspase-9 activity (Figure 2B) increased over 4-6 folds after XL388 treatment (250 nM, 24h). Furthermore, cleavages of caspase-3, caspase-9 and PARP were detected in XL388-treated A172 cells (Figure 2C). Following XL388 treatment, mitochondrial depolarization was detected in A172 cells, which was evidenced by accumulation of JC-1 green monomers (Figure 2D). Further studies demonstrated that TUNEL-positive cell nuclei ratio was significantly increased following XL388 treatment in A172 cells (Figure 2E). Cell apoptosis was further supported by a significant increase of Annexin V-positive staining in XL388-treated cells (Figure 2F). In U251 cells and Pri-1/Pri-2 primary glioma cells, XL388 similarly increased caspase-3 activity (Figure 2G) and induced apoptosis activation (TUNEL assays, Figure 2H). Significantly, XL388, at 250 nM, failed to inhibit cell viability (Figure 2I) and induce cell apoptosis (Figure 2J) in primary human astrocytes (“Astrocytes”) and HCN-1a neuronal cells. Both are non-cancerous cells [12, 13]. These results demonstrated that XL388 induced significant apoptosis activation in glioma cells.

### XL388-induced anti-glioma cell activity is through Akt-mTOR-dependent and -independent mechanisms

**Figure 3:**
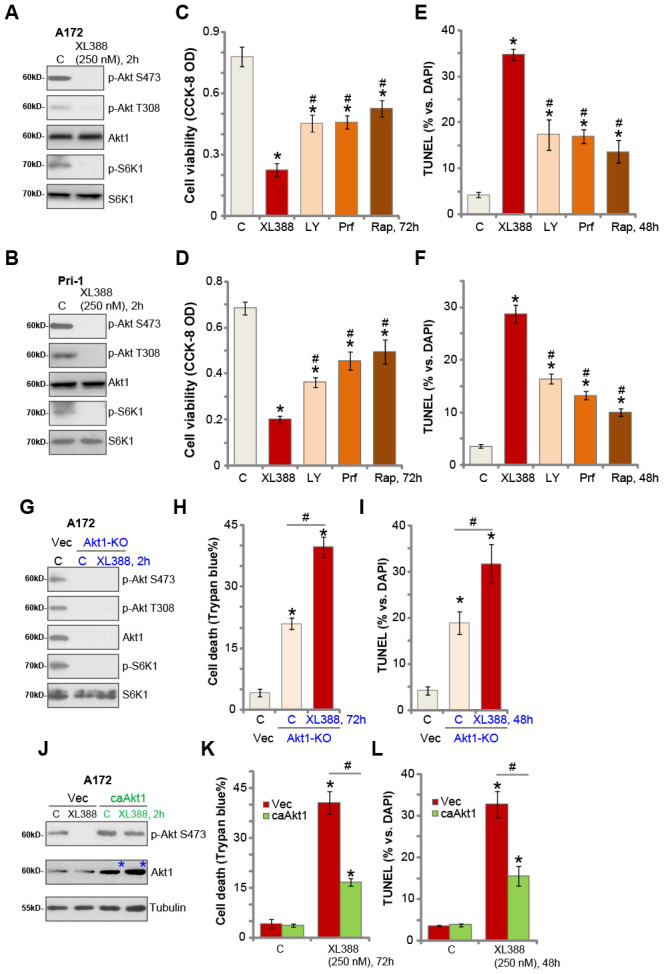
**XL388-induced anti-glioma cell activity is through Akt-mTOR-dependent and -independent mechanisms.** A172 cells or the primary human glioma cells, Pri-1, were treated with XL388 (250 nM), and cultured for 2h, and expression of listed proteins was shown (**A** and **B**). A172 cells or the Pri-1 primary human glioma cells were treated with XL388 (250 nM), LY294002 (“LY”, 1 μM), perifosine (“Prf”, 5 μM) or rapamycin (“Rap”, 500 nM) for 48-72h, then cell viability and apoptosis were tested by CCK-8 (**C** and **D**) and TUNEL staining (**E** and **F**) assays, respectively. Stable A172 cells with the CRISPR/Cas9-Akt1-KO construct (“Akt1-KO” cells) or empty vector (“Vec”) were treated with or without XL388 (250 nM) for applied time, and cultured for applied time periods, and expression of listed proteins was shown (**G**);Cell death and apoptosis were tested by Trypan blue staining (**H**) and nuclear TUNEL staining (**I**) assays, respectively. Stable A172 cells with a constitutive-active Akt1 (S473D, “ca-Akt1”) or empty vector (“Vec”) were treated with or without XL388 (250 nM) for applied time, and expression of listed proteins was shown (**J**); cell death and apoptosis were tested by Trypan blue staining (**K**) and nuclear TUNEL staining (**L**) assays, respectively. Data were presented as mean ± SD (n=5).* p <0.05 vs. “C” cells. ^#^p <0.05 vs. XL388 treatment (**C**–**F**). ^#^p <0.05 (**H**, **I**, **K** and **L**). Experiments in this figure were repeated three times, and similar results were obtained.

XL388 is a PI3K-mTOR dual inhibitor [16, 24]. Its effect on mTOR signaling in glioma cells was studied next. Western blotting results in A172 cells demonstrated that XL388 treatment (250 nM, 2h) blocked activation of mTORC1 and mTORC2, which were reflected by phosphorylated (p-) S6K1 and p-Akt Ser-473, respectively (Figure 3A) [11, 25, 26]. Activation of Akt, tested by p-Akt at Ser-473 and Thr-308, was blocked by XL388 as well (Figure 3A). In primary glioma cells, Pri-1, the PI3K-mTOR dual inhibitor blocked Akt-mTORC1/2 activation (Figure 3B). We compared the activity of XL388 with other known Akt-mTOR inhibitors, including the pan Akt-mTOR inhibitor LY294002 [27], the Akt specific inhibitor perifosine [28], the mTORC1 inhibitor rapamycin. In A172 cells and Pri-1 glioma cells, XL388 (250 nM)-induced viability reduction (Figure 3C and 3D) and apoptosis (Figure 3E and 3F) were significantly more potent than LY294002, perifosine and rapamycin. Notably, the three were utilized at higher concentrations than XL388.

These results suggest that Akt-mTOR-independent mechanisms could also be responsible for XL388-induced activity in glioma cells. To test this hypothesis, the CRISPR/Cas9 strategy was applied to knockout Akt1. As shown, Akt1 is completely depleted in stable A172 cells (“Akt1-KO” cells) with the CRISPR/Cas9-Akt1-KO construct (from Dr. Zhang at Soochow University [29]) (Figure 3G), showing Akt-mTORC1/2 blockage (Figure 3G). Importantly, in the Akt1-KO cells XL388 was able to induce further cell death (Figure 3H) and apoptosis (Figure 3I).

Next, a constitutive-active Akt1 (ca-Akt1, S473D, from Dr. Fang at Shanghai Jiao Tong University [30]) was transduced to A172 cells and stable cells were established with GFP sorting (Figure 3J, blue star). As shown, ca-Akt1 restored Akt activation even after XL388 treatment (250 nM, 2h) in A172 cells (Figure 3J). Importantly, ca-Akt1 only partially inhibited, but not reversed, XL388-induced A172 cell death (Figure 3K) and apoptosis (Figure 3L). Therefore, XL388 induces significant cytotoxicity in glioma cells through Akt-mTOR-dependent and -independent mechanisms.

### XL388 induces oxidative injury in human glioma cells

**Figure 4:**
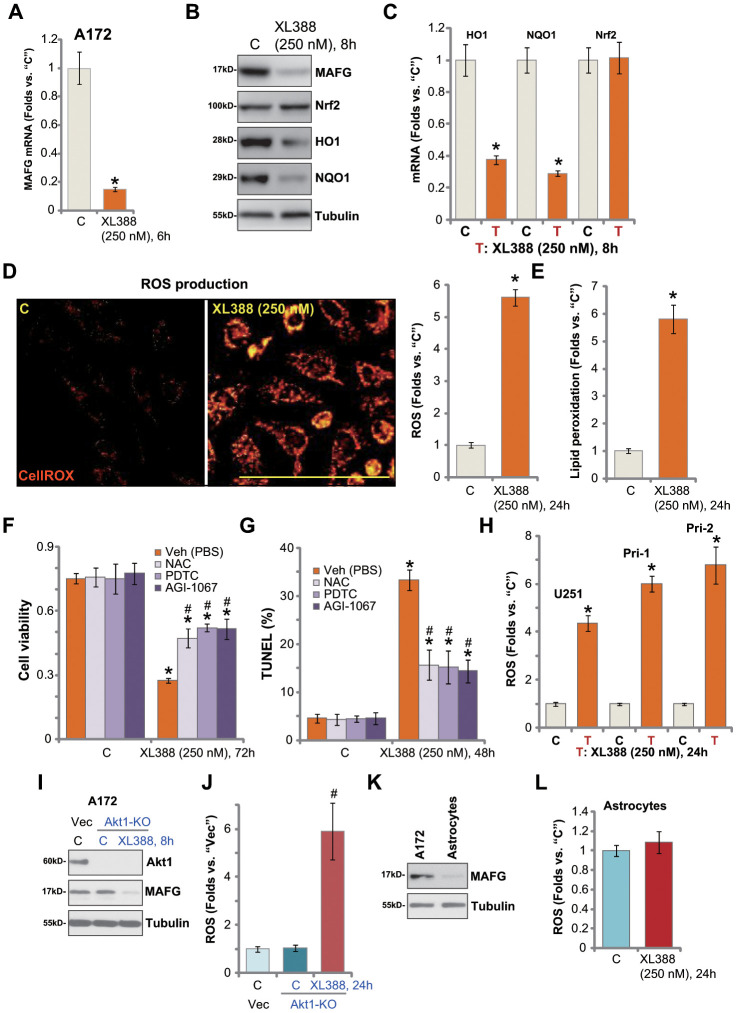
**XL388 induces oxidative injury in human glioma cells.** A172 cells or primary human glioma cells (“Pri-1”) were treated with XL388 (250 nM) and cultured for indicated time periods, then expression of listed mRNAs and proteins was tested by qPCR and Western blotting assays (**A**–**C**); Relative CellROX intensity (**D**) and lipid peroxidation (**E**) levels were tested. A172 cells were pretreated for 1h with n-acetylcysteine (NAC, 400 μM), pyrrolidine dithiocarbamate (PDTC, 10 μM) or AGI-1067 (10 μM), followed by XL388 (250 nM) stimulation for another 48-72h, then cell viability and apoptosis were tested by CCK-8 (**F**) and nuclear TUNEL staining (**G**) assays, respectively. U251MG (“U251”) and primary human glioma cells (“Pri-1/Pri-2”) were treated with XL388 (250 nM) for 12h, then the relative CellROX intensity was tested (**H**). A172 cells with the CRISPR/Cas9-Akt1-KO construct (“Akt1-KO” cells) or empty vector (“Vec”) were treated with or without XL388 (250 nM) and expression of listed proteins was shown (**I**). Relative ROS contents were tested by measuring CellROX intensity (**J**). Expression of MAFG protein in A172 cells and primary human astrocytes (“Astrocytes”) was shown (**K**); Astrocytes were treated with or without XL388 (250 nM) for 24h, and ROS intensity tested by CellROX assay (**L**). Data were presented as mean ± SD (n=5).* p <0.05 vs. “C” cells. ^#^p <0.05. “Veh”-pretreated cells (F, G and J). Experiments in this figure were repeated three times, and similar results were obtained. Bar= 100 μm (**D**).

Studies have shown that MAFG associates with Nrf2 in cell nuclei [31], required for Nrf2 transcriptional activation and expression of anti-oxidant genes [31, 32]. In A172 glioma cells, *MAFG* mRNA expression was significantly downregulated following XL388 treatment (Figure 4A). MAFG protein level was decreased as well (Figure 4B). As a result, expression of Nrf2-dependent mRNAs, *HO1* and*NQO1*, was reduced (Figure 4C), and HO1-NQO1 protein downregulated as well (Figure 4B). Interestingly, *Nrf2* mRNA and protein expression was unchanged after XL388 treatment (Figure 4B and 4C). Thus, XL388 downregulated MAFG and inhibited Nrf2 signaling in A172 cells.

Inhibition of Nrf2 signaling could lead to ROS production and oxidative injury [21, 33, 34]. By measuring CellROX fluorescence [35, 36], we show that XL388 induced significant ROS production in A172 cells (Figure 4D). Furthermore, the cellular lipid peroxidation levels were significantly increased (Figure 4E). To test the link between XL388-induced oxidative stress and glioma cell death, ROS scavengers were utilized, including n-acetylcysteine (NAC), pyrrolidine dithiocarbamate (PDTC) [37] and AGI-1067 [38, 39]. As shown, NAC, PDTC or AGI-1067 alleviated XL388-induced viability reduction (Figure 4F) and cell apoptosis (TUNEL assays, Figure 4G). These results indicated that ROS production and oxidative injury participated in XL388-induced cytotoxicity in A172 cells. In U251 cells and primary glioma cells (Pri-1/Pri-2), XL388 similarly induced oxidative injury and increased CellROX intensity (Figure 4H).

In A172 cells, Akt1 KO (see Figure 3) failed to downregulate MAFG expression (Figure 4I) or inducing ROS production (CellROX intensity, Figure 4J). However, XL388 induced MAFG downregulation (Figure 4I) and significant ROS production (Figure 4J) in the Akt1-KO A172 cells. These results further supported that XL388-induced MAFG downregulation and ROS production are independent of Akt inhibition in glioma cells. In the primary human astrocytes, MAGF expression is low (Figure 4K). XL388 treatment had little effect on ROS in astrocytes (Figure 4L). This could also explain why XL388 is ineffective against astrocytes (Figure 2I and 2J).

### XL388 oral administration inhibits A172 xenograft growth in severe combined immunodeficient (SCID) mice

**Figure 5:**
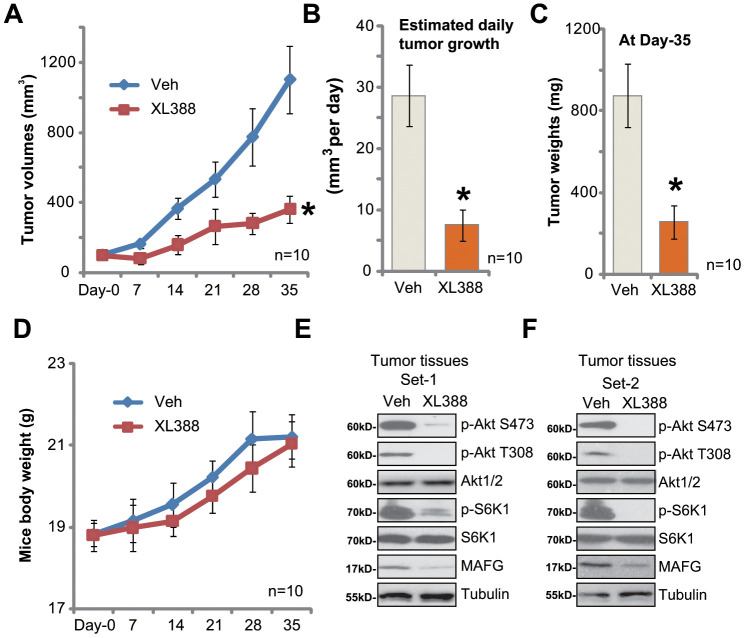
**XL388 oral administration inhibits A172 xenograft growth in SCID mice.** The SCID mice bearing A172 xenografts (n=10 per group) were administrated with vehicle (saline, “Veh”) or XL388(5 mg/kg body weight, daily, × 14d), then tumor volumes (in mm^3^) (**A**) and mice body weights (in grams) (**D**) was recorded every seven days for a total of 35 days; The estimated daily tumor growth (in mm^3^ per day) was calculated as described (**B**). At treatment Day-35, all tumors were isolated and individually weighted (**C**). At treatment Day-7, two hours after initial XL388administration, the xenograft tumors were isolated. Tissue lysates were subjected to Western blotting assays of listed proteins (**E** and **F**). *p < 0.05 vs. “Veh” group (**A**–**C**).

As described in our previous studies [12, 13], A172 glioma cells were injected *s.c.* to the SCID mice. The A172 tumor xenografts were established within two weeks (volume of each tumor around 100 mm^3^, “Day-0”). The tumor-bearing SCID mice were treated with XL388 or the vehicle control. As shown, oral administration of XL388 (5 mg/kg body weight, daily, × 14d) [16, 24] potently inhibited A172 xenograft growth in SCID mice (Figure 5A). Volumes of XL388-treated A172 xenografts were lower than those of vehicle control tumors (Figure 5A). The estimated daily tumor growth, calculated by the formula: (Tumor volume at Day-35—Tumor volume at Day-0)/35, again demonstrated that XL388 significantly inhibited A172 xenograft growth (Figure 5B). At Day-35, all tumors were isolated and weighted individually. XL388-treated A172 xenografts were significantly lighter than the vehicle tumors (Figure 5C). Thus, XL388 oral administration inhibited A172 xenograft growth in SCID mice. The mice body weights were not significantly different between the vehicle group and XL388 treatment group (Figure 5D).

At treatment Day-7, two hours afterXL388 or vehicle administration, two xenografts of each group were isolated and tissue lysates were achieved. Results in Figure 5E and 5F confirmed that phosphorylation of Akt and S6K1 was largely inhibited in XL388-treated tumors, indicating Akt-mTORC1/2 inactivation. Furthermore, MAFG downregulation was detected (Figure 5E and 5F).

## DISCUSSION

Glioma is the most common primary tumor in central never system and is among the most aggressive of all human malignancies [1, 2]. Overactivation of Akt-mTOR cascade is frequently detected in human glioma, promoting tumor cell survival, growth, proliferation, motility, angiogenesis and apoptosis-resistance [40–42]. Small molecule inhibitors of Akt-mTOR cascade have exhibited favorable preclinical results and entered clinical trials for human glioma [40, 41]. However, the limited single-agent activity of rapamycin analogs in several glioma trials [43, 44] provides a rationale for further testing other Akt-mTOR inhibitors against human glioma [40, 41].

Here we show that XL388 blocked Akt-mTORC1/2 activation in established and primary human glioma cells. XL388 potently inhibited glioma cell viability and proliferation as well as cell migration, invasion and cell cycle progression. The PI3K-mTOR dual inhibitor induced significant apoptosis activation in glioma cells. Significantly, oral administration of XL388 potently inhibited A172 xenograft growth in SCID mice. These results indicated that XL388 might have important therapeutic value for human glioma.

Although XL388 blocked Akt-mTORC1/2 activation, XL388-induced cytotoxicity in glioma cells is not solely dependent on Akt-mTORC1/2 inhibition. First, XL388 is significantly more potent than other known Akt-mTOR inhibitors (LY294002, perifosine and rapamycin) in killing glioma cells. Second, restoring Akt-mTOR activation, by caAkt1, only partially attenuated XL388-induced glioma cell death. Third, XL388 is yet still cytotoxic and pro-apoptotic in Akt1-KO A172 cells. These results confirmed the co-existence of Akt-mTOR-independent mechanisms responsible for XL388-induced anti-glioma cell activity. Indeed, we show that MAFG-Nrf2 inhibition could be another mechanism for XL388-induced actions in glioma cells.

Emerging studies have proposed that MAFG could be an important oncogenic gene for tumorigenesis and progression [45]. Liu et al.*,* showed that MAGF is overexpressed in hepatocellular carcinoma (HCC), associated with tumor progression and reduced survival time [45]. Vera-Puente et al.*,* proposed that MAFG is a potential therapeutic target of non-small-cell lung cancer (NSCLC). MAFG silencing increased ROS production to sensitize cancer cells cisplatin [32]. Fang and colleagues demonstrated that BRAF^V600E^-stabilized MAFG initiated recruitment of a co-repressor complex to CpG island methylator phenotype (CIMP) gene promoters. It will then promote tumorigenesis in colorectal cancer [46]. Conversely, MAFG silencing inhibited CRC cell growth [46].

We showed that XL388 downregulated MAFG and inhibited Nrf2 signaling, causing significant ROS production and oxidative injury in glioma cells. Several antioxidants, including NAC, PDTC and AGI-1067, alleviated XL388-induced glioma cell death and apoptosis. Importantly, MAFG expression was unchanged in Akt-KO A172 cells. These results indicated that MAGF downregulation could be an unique action of XL388, which is responsible for the superior anti-glioma cell activity by this compound.

The current *in vitro* results and subcutaneous xenograft studies could not be directly translated to humans, and thus the efficacy and safety of XL388 against human glioma will definitely need further characterizations. Testing this compound at lower concentrations in an in-situ glioma xenograft model is certainly needed in the following studies. The underlying signaling mechanisms of XL388-induced MAFG downregulation and Nrf2 inhibition warrant additional studies as well.

## MATERIALS AND METHODS

### Chemicals and reagents

XL388 was provided by Dr. Zhang [24] at Southeast University of China. N-acetylcysteine (NAC), pyrrolidine dithiocarbamate (PDTC), AGI-1067, puromycin, rapamycin, perifosine and LY294002 were obtained from Sigma-Aldrich (St. Louis, MO). Cell culture reagents were purchased from Gibco-BRL Co. (Grand Island, NY). Antibodies utilized in this study were provided by Cell Signaling Tech (Shanghai, China) and Abcam Co. (Beijing, China). TRIzol and other reagents for RNA assays as well as Lipofectamine 2000 and other transfection reagents were provided by Thermo-Fisher Invitrogen Co. (Shanghai, China).

### Cell culture

Cultures of established glioma cell lines, A172 and U251MG, human neuronal HCN-1a cells, as well as the primary human astrocytes and primary human glioma cells (“Pri-1/Pri-2”, derived from two primary glioma patients) were described in detail in our previous studies early [12–14, 47]. The protocols of this study were approved by the Ethics Review Board (ERB) of Shanghai Jiao-Tong University School of Medicine, according to the principles of Declaration of Helsinki.

### Quantitative real-time reverse transcriptase polymerase chain reaction (qPCR)

The detailed protocols for qPCR, using SYBR Master Mix and the ABI Prism 7500H Fast Real-Time PCR system, were described early [12, 13]. Quantization of target mRNA was through the 2^—ΔCt^ method. The mRNA primers of *humanNrf2*, *HO1*, *NQO1* and *GAPDH* were described previously [48]. The mRNA primers of *human MAFG* were purchased from Origene (Beijing, China).

### Constitutively-active mutant Akt1

The recombinant adenovirus expressing constitutively-active Akt1(caAkt1, S473D, with GFP tag) construct was a gift from Dr. Fang at Shanghai Jiao Tong University [30]. caAkt1 adenovirus or the empty vector adenovirus (Ad-GFP) was added to A172 cells. The infected cells expressing GFP were sorted by FACS and stable cells established. Expression of caAkt1 was verified by Western blotting.

### Akt1 knockout

A lenti-CRISPR-GFP Akt1-knockout (KO) construct was from Dr. Zhang at Soochow University [29]. A172 cells were cultured into six well plates at 60% confluence, transfected with the Akt1-KO construct. The transfected cells with GFP were sorted by FACS, and stable single cells were established. Akt1 KO was verified by Western blotting.

### Cell Counting Kit-8 (CCK-8) viability assay

Cells were plated at a density of 3 ×10^3^ cells/well into 96-well plates. Following the applied treatments, the CCK-8 (10μL/well, MCE, Shanghai, China) dye was added, and cells were further incubated for additional 2h. CCK-8 optical density (OD) was tested at 450 nm.

### Colony formation assay

A172 glioma cells (5, 000 cells for each treatment) were resuspended in agar (0.5%, Sigma)-containing complete medium (with 10% FBS), added on the top of 10-cm culture dishes. XL388-contianing medium or the vehicle control medium was renewed every two days for 10 days. Afterwards, A172 colonies were stained and manually counted.

### EdU staining assay of cell proliferation

C**ells** were seeded into six-well plates at 1 × 10^5^ cells per well. Following the applied treatments, an EdU (5-ethynyl-20-deoxyuridine) Apollo-567 Kit (RiboBio, Guangzhou, China) was utilized to quantify cell proliferation. EdU and DAPI were both added to the cultured cells, and visualized under a fluorescent microscope. EdU ratio (% vs. DAPI) was calculated.

### Apoptosis and cell cycle assays

Cell apoptosis was tested by Annexin V FACS, nuclear TUNEL staining and caspase-3/caspase-9 activity assays. The detailed protocols were described in previous studies [49, 50]. Propidium Iodide (PI)-FACS assay of cell cycle progression was described early [51].

### Cell death detection by trypan blue staining

Following the applied treatment, trypan blue was added to stain the “dead” glioma cells. Cell death percentage was calculated by the automated cell counter (Merck Millipore, Soochow, China), and the Trypan blue ratio was recorded.

### Mitochondrial depolarization assay

JC-1, a mitochondrial fluorescence dye, will aggregate in the mitochondrial inner membrane of stressed cells with mitochondrial depolarization, forming green monomers [52]. Glioma cells were seeded into 24-well plates at 50-60% of confluence, and treated with XL388. Afterwards, cells were stained with JC-1 (10 μg/mL, Sigma), washed and tested under a fluorescence spectrofluorometer (F-7000, Hitachi, Japan) at test-wavelength of 488 nm (green). The representative JC-1 images, integrating both green fluorescence (at 488 nm) and red fluorescence (at 625 nm), were presented as well.

### I*n vitro* migration and invasion assays

As described early [12], for each treatment 3 × 10^4^ glioma cells were seeded onto the upper surface of the “Transwell” chambers (12-μm pore size, BD Biosciences, Shanghai, China). The lower compartments were filled with complete medium (with 10% FBS). After incubation for 16h, the non-migrated cells on the upper surfaces were removed, and on the lower surfaces the migrated cells were fixed, stained and counted. To test cell invasion, “Transwell” chambers were coated with Matrigel (Sigma, Shanghai, China).

### Western blotting

Western blotting protocol was described in our previous studies [12, 53, 54]. Note that the same set of lysates were run in sister gels to test different proteins.

### Lipid peroxidation

Using a previously-described protocol [55] cellular lipid peroxidation level was analyzed. In brief, A172 cells were seeded (1 × 10^5^ cells per well into six-well plates). Following XL388 treatment, a lipid peroxidation assay kit (Abcam, Shanghai, China) was applied to quantitatively measure cellular lipid peroxidation intensity, tested by the thiobarbituric acid reactive (TBAR) concentration through the described protocols [55, 56].

### ROS detection

Cells were seeded into six-well plates at 1 × 10^5^ cells per well. Following the applied treatments, cells were stained with CellROX dye for 30 min under the dark. The red fluorescence (at 625 nm) was detected and representative CellROX images were shown.

### Xenograft assay

As previously reported [12, 13], the female severe combined immunodeficient (SCID) mice were purchased from The Animal Center of Soochow University (Suzhou, China) and housed under the standard procedures. A172 cells (5×10^6^ cells of each mice in 200 μl of Matrigel gel, no serum) were subcutaneously (*s.c.*) injected to the flanks of the SCID. In two weeks with the volume reaching approximately 100 mm^3^ for each tumor (“Day-0”), mice were randomly assigned into two groups with 10 mice per group. Tumor volumes were calculated as described [12, 13]. All animal procedures were approved by IACUC of Shanghai Jiao-Tong University School of Medicine.

### Statistical analyses

In this study, statistics were calculated by using SPSS 23.0 software (SPSS Co., Chicago, IL). Descriptive statistics including mean and standard deviation (SD) along with one-way ANOVAs were applied to determine significant differences. A Two-tailed unpaired T test (Excel 2013) was utilized to test significance between two treatment groups. P values < 0.05 were considered significant.
